# THE EFFECTIVENESS OF FORAMINAL ROOT BLOCK IN RELIEVING SCIATIC PAIN DUE TO LUMBAR DISC HERNIATION

**DOI:** 10.1590/1413-785220233105e263169

**Published:** 2023-10-23

**Authors:** ÂNGELO SANTANA GUERRA, MARCELLO OLIVEIRA BARBOSA, MATHEUS MORENO DE OLIVEIRA, ROSANA QUEIROZ COCCOLI, ANGELO AUGUSTO BONGIOLO GANEO, EDUARDO JOSÉ FERREIRA SALES

**Affiliations:** 1 Hospital de Base do Distrito Federal, Brasília, DF, Brasil.; 2 Hospital Regional do Paranoá, Brasília, DF, Brasil.; 3 Hospital Regional do Gama, Brasília, DF, Brasil.; 4 Hospital Regional de Taguatinga, Brasília, DF, Brasil.

**Keywords:** Disc Herniation, Nerve Block, Sciatica Neuropathy, Hérnia de Disco, Bloqueio Nervoso, Neuropatia Ciática

## Abstract

**Objective:**

To evaluate the clinical profile, pain improvement, and the need for surgical interventions in patients undergoing transforaminal block with the use of corticosteroids and anesthetics.

**Methods:**

This is a prospective, randomized, double-blind study with 45 patients with unilateral radicular pain in their lower limbs and a single-segment lumbar disc herniation diagnosis. In the intervention group, transforaminal blocks with bupivacaine, dexamethasone, and clonidine were applied and in the control group, distilled water and bupivacaine. The Oswestry questionnaire was applied.

**Results:**

We included 24 female (53.4%) and 21 male patients (46.6%). Of those with an occupation, 85.71% (n = 30) were relieved from their duties due to their illness and 14.29% (n = 5) continued to work with limitations. Those who underwent transforaminal block with an injection of corticosteroids, clonidine, and anesthetics showed immediate relief. However, such effect failed to alleviate patients’ symptoms after three weeks. We observed that 52% of patients showed varying degrees of improvement. The control group experienced mild pain relief after one week, which also failed to last after three weeks. Moreover, 50% of patients improved in varying degrees.

**Conclusion:**

Further studies with larger samples, new epidemiological data, and longer follow-ups are necessary to validate our hypotheses. **Level of Evidence II, Prospective Study.**

## RESUMO

**Objetivo:**

Avaliar o perfil clínico, a melhora da dor e a necessidade de intervenções cirúrgicas em pacientes submetidos ao bloqueio transforaminal com uso de corticosteroides e anestésicos.

**Métodos:**

Estudo prospectivo, randomizado e duplo-cego realizado com 45 pacientes com dor radicular unilateral em membros inferiores e diagnóstico de hérnia discal lombar em um único segmento. No grupo intervenção, os bloqueios transforaminais foram feitos com bupivacaína, dexametasona e clonidina; no controle, água destilada e bupivacaína. Foi aplicado questionário de Oswestry.

**Resultados:**

A amostra foi composta de 24 mulheres (53,4%) e 21 homens (46,6%). Dos pacientes com ocupação, 85,71% (n = 30) estavam afastados de suas funções devido à doença e 14,29% (n = 5) continuavam a trabalhar com limitações. Os que foram submetidos ao bloqueio transforaminal com injeção de corticoide, clonidina e anestésico apresentaram alívio imediato. Após três semanas, contudo, o efeito não perdurou de forma tão satisfatória, e 52% dos pacientes apresentaram melhora em graus variados. No grupo controle, houve discreto alívio álgico após uma semana, que não perdurou de forma satisfatória após três semanas, com 50% dos pacientes evoluindo para melhora em graus variados.

**Conclusão:**

Mais estudos com espaço amostral maior, novos dados epidemiológicos e seguimento mais prolongado são necessários para validar as hipóteses aventadas. **Nível de Evidência II, Estudo Prospectivo.**

## INTRODUCTION

Chronic pain configures a public health issue that may be associated with trauma or illness and persist after the initial injury has healed.^1^ This condition generates a growing demand for public health services and includes prolonged treatments and financial impacts.^2^

The current aging of the population has increased the prevalence of chronic and degenerative diseases and the incidence of pain and disability. Patients mainly complain of chronic pain, which markedly interferes with their quality of life.^2^ ^),(^ ^3^ Low back pain, one of the most common health problems in adults, refers to pain and discomfort below individuals’ costal margin and above their upper gluteal line, which may include pain in lower limbs and be classified as chronic if it persists for more than three months.^1^

Lumbar disc herniation is the most common diagnosis among the degenerative changes to the lumbar spine, configuring one of the main conditions causing chronic pain and one of the biggest causes of sickness benefits in Brazil due to disability retirement and surgical intervention, according to INSS 2019 data.^4^ ^)-(^ ^6^ It shows a multifactorial etiology related to occupations with physical exertion and high workloads,^7^ often occurring between individuals’ third and fifth decades of life (the mean age of the first acute episode revolves around 37 years). Estimates suggest that from 2 to 3% of the population have a diagnosis of lumbar disc herniation (especially in people aged over 35 years) and a 4.8 and 2.5% prevalence in men and women, respectively.^4^ ^),(^ ^5^ ^),(^ ^7^ ^),(^ ^8^

Disc herniation consists of the displacement of the nucleus pulposus in an intervertebral disc due to a rupture of the annulus fibrosus - usually in its posterolateral region.^3^ ^),(^ ^4^ ^),(^ ^8^ ^)-(^ ^10^ Its clinical manifestations will depend on the volume of herniated material due to the compression and irritation of lumbar roots and dural sac. Its symptomatology includes initial low back pain, which may evolve to radicular pain, lumbosciatalgia (accompanying the dermatome corresponding to the compromised level, radiating to the gluteus or posterior thigh, and changing with the movement of the lumbar spine), or pure sciatica.^3^ ^)-(^ ^5^ ^),(^ ^8^ ^),(^ ^11^

Proper physical examination is essential for diagnosis, which can determine the vertebral space in which the hernia is located by carefully evaluating dermatomes and myotomes. Some specific tests, such as Lasègue’s sign and hip extension, can help diagnoses by reproducing or increasing patients’ pain. Imaging tests are also critical for the diagnosis, location, classification, and prognosis of the disease, including magnetic resonance imaging, the gold standard for diagnosis.^4^ ^).(^ ^5^

Therapy prioritizes minimally invasive surgical procedures due to their lower tissue aggression, shorter hospitalization times, lower anesthetic risks, and early return to work activities.^4^ ^),(^ ^12^ Radioscopy-guided transforaminal and epidural blocks exemplify such minimally invasive techniques to treat lumbar disc herniation.^4^ ^),(^ ^12^ ^),(^ ^13^

This study aimed to evaluate the clinical profile, pain improvement, and the need for surgical interventions in patients undergoing these minimally invasive techniques, especially transforaminal blocks with corticosteroids.

## METHODS

### Study design

This is a prospective, randomized, double-blind study in which 45 patients with unilateral radicular pain in their lower limbs and evidence of single-segment lumbar disc herniation were evaluated from November 2018 to April 2020.

### Ethical aspects

Informed consent forms describing the risks and benefits of the instituted therapy were signed by all patients, who were allocated into two groups by coin tosses: “heads” for group 1 (intervention) and “tails” for group 2 (control). This study was approved by the National Research Ethics Committee under number 3.104.615/18 (available on Plataforma Brasil).

### Exclusion criteria

Presence of other diseases with pain symptoms (such as trochanteric bursitis, gluteal tendinopathy, and coxarthrosis), lumbar tumors, infections with root compression, diagnosed renal lithiasis, patients who used anticoagulants or antiplatelet agents; those who had undergone a foraminal and/or epidural block in the previous three years; and those who had undergone disc surgical procedures such as microdiscectomy, open discectomy, or arthrodesis.

### Performed technique

Patients were positioned in horizontal ventral decubitus, their abdomen was supported by a pillow, their hips and knees were semi-flexed at about 30 degrees, and their vital parameters were monitored.

**Figure 1:**
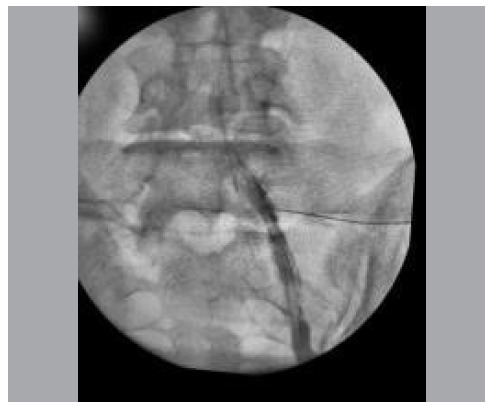
Nerve root delineated by contrast and visualized on radioscopy.

A supported surgical instrument under patients’ lumbar region was used to establish the desired exact target. The tip of the instrument was mobilized until it coincided with the target of an image corresponding to the relevant lower pedicle under a slight medial deviation. The target was locally infiltrated with 2% lidocaine. A 22-gauge 3.5-inch spinal needle with a Quincke tip was introduced following the coaxial technique and observed as a single point under radioscopy along its path (Figure 1).

As the needle was introduced, the position of the radioscopy equipment was changed to provide the incidence of the absolute profile so the needle depth could be controlled. A contrast was injected under continuous radioscopy to monitor its distribution in the epidural space and ensure adequate infiltration into the extravascular medium. A solution was injected after we obtained an adequate distribution and good anatomical delimitation according to the contrast. Finally, patients were referred for post-anesthetic recovery and observation for 30 minutes. No medication was prescribed after the procedure.

In the intervention group, block solutions were composed of 1 mL of 0.5% bupivacaine (a local anesthetic), 2 mL of dexamethasone 10 mg/2.5 mL, and 1 mL of clonidine 150 mcg/mL. In the control group, blocks were performed with 3 mL of distilled water and 1 mL of 0.5% bupivacaine.

A simplified Oswestry disability questionnaire was applied before the procedure (Q1), one week after it (Q2), and three weeks after it (Q3). It consisted of 10 graded questions (from 0 to 5 points) on pain intensity; personal care; ability to carry load, move, and remain in a position for a certain time; changes in sleep, sexual and social life; and inability to travel. The closer to 100%, the greater patients’ disability and the closer to 0%, the lower patients’ disability.

## RESULTS

In total, 45 patients participated in this study, 53.4% of which were women and 46.6% men. Mean female and male age totaled 51.1 years (SD=10.65) and 49.1 (SD=9.75), respectively. The intervention group consisted of 25 patients, mostly women (60%; n = 15) with a mean age of 52.2 years (± 10.8). The control group included 20 patients, 55% of which were men (n = 11) with a mean age of 50.4 years (± 8.8) (Table 1).

**Table 1:** Demographic data

	Intervention (n = 25)	Control (n = 20)	Total (n=45)
Age	51.25 (± 10.25)	48.8 (± 10.38)	50.23 (± 10.31)
Gender			
Male	10 (40%)	11 (55%)	21 (46.7%)
Female	15 (60%)	9 (45%)	24 (53.3%)

About 77.78% of our patients had a salaried occupation and 8.89% of them had retired before showing typical lumbar compression. Of the patients who had an occupation, 85.71% were on sick leave and 14.29% continued to work with limitations. Only six patients reported no paid activities. Domestic workers (22.9%) and bricklayers/construction assistants (17.1%) were the most prevalent occupations (Table 2).

**Table 2:** Occupational data.

Occupation	Total (n=35)	On sick leave (n = 30)
Domestic worker	8	7
Bricklayer/Construction assistant	6	6
General services	5	4
Trader	4	1
Electrician	2	2
Administrative worker	2	2
Teacher	2	2
Laundry worker	1	1
Attendant	1	1
Marble & granite worker	1	1
Carpenter	1	1
Nursing Technician	1	1
Psychopedagogue	1	1

About 80% of patients underwent one or more therapeutic modalities, such as physical therapy, hydrotherapy, and acupuncture. We found that 76% of patients in the intervention group underwent physical therapy; 25% hydrotherapy; and 28%, acupuncture; whereas in the control group, 75% underwent physical therapy; 20%, hydrotherapy; 45% acupuncture (Table 3). No evaluated cases evinced that these adjuvant therapies had satisfactorily and regularly relieved patients’ symptoms.

**Table 3:** Adjuvant treatments.

	Intervention (n = 25)	Control (n = 20)	Total (n=45)	
Adjuvant Treatment	20 (80%)	16 (80%)	36	(80%)
Physical therapy	19 (76%)	15 (75%)	34	(75.55%)
Hydrotherapy	5 (25%)	5 (20%)	10	(22.22%)
Acupuncture	7 (28%)	9 (45%)	16	(35.55%)

The most frequent block level (Table 4) in our sample was L5-S1 (53.3%), followed by L4-L5 (40%) and L3-L4 (6.7%). Overall, blocks on patients’ right side prevailed (64.4%). The control group showed a predominance of L5-S1 (50%) blocks on patients’ right side (75%). The intervention group showed a similar predominance of L5-S1 blocks (56% of our sample) on volunteers’ right side (56% of all blocks).

**Table 4:** Block level and side.

	Intervention (n = 25)	Control (n = 20)	Total (n=45)
Nerve roots			
L3-L4	2 (8%)	1 (5%)	3 (6.7%)
L4-L5	9 (36%)	9 (45%)	18 (40%)
L5-S1	14 (56%)	10 (50%)	24
Affected side			
Left	11 (44%)	5 (25%)	16 (35.6%)
Right	14 (56%)	15 (75%)	29 (64.4%)

To better understand patients’ improvement or worsening, after summing and comparing their Q1, Q2, and Q3 questionnaire scoring, we divided volunteers into groups according to varying degrees of improvement or worsening over one or three weeks after the procedure. The +A group represented those patients whose condition improved as their scores showed a decrease of about 20 points between Q1 and Q2 or Q3 (i.e., over one or three weeks after the block), whereas the −A Group represented those whose questionnaire scores showed an increase of 20 points (i.e., in which patients’ symptoms significantly worsened over one or three weeks). Subsequent groups (+B/−B; +C/−C; +D/−D) followed the same order. The B Group included all scores that varied from 10 to 19 points; the C Group, from five to nine points; and the D Group, from one to four points. Patients in the A Group show large variations between the symptomatology reported at the time of the block and subsequent scenarios; whereas those in the B and C Groups, moderately so; and those in the D Group, very little.

**Figure 2:**
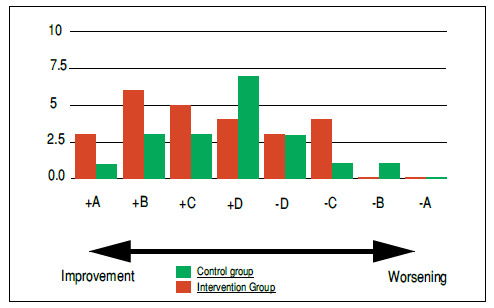
Quantitative comparison of patients who reported pain improvement or worsening one week after undergoing blocks for the intervention (red) and control groups (green).

Regarding our evaluation of the variation between pre-block questionnaire scores and those one week after the procedure, Figure 2 shows that the intervention group had three patients (12%) in the +A group, six (24%) in the +B group; five (20%) in the +C group, and four (16%) in the +D group; whereas the intervention group patients with higher questionnaire scores (reflecting clinical worsening) referred to three volunteers (12%) in the −D group, four (16%) in the −C group, and no participants in the −B and −A groups.

We found that one patient in the control group (5%) was in the +A group, three (15%) in the +B group, three (15%) in the +C group, and seven (35%) in the +D group; whereas the patients in this group who experienced symptom worsening in the first week after the procedure included three patients (15%) in the −D Group, one (5%) in the −C Group, one (5%) in the −B Group and no volunteer in the −A Group. Finally, one patient (5%) showed no variation between Q1 and Q2.

**Figure 3:**
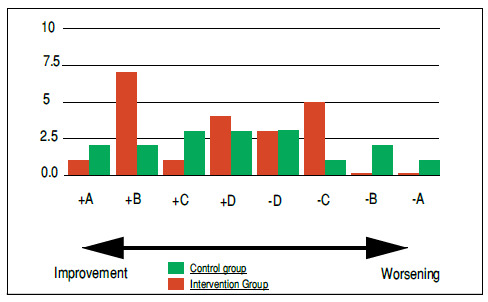
Quantitative comparison of patients who reported improved or worsened pain three weeks after undergoing blocks in the intervention (red) and control groups (green).

Figure 3 shows our comparative analysis between pre-block scores and those three weeks after the procedure. After three weeks, we included one intervention group patient (4%) in the +A group; seven (28%) in the +B group; one (4%) in the +C group; and four (16%) in the +D group. In the same group, of those patients who showed worsened pain and limitations, three (12%) were in the −D group; five (20%), in the −C group, and no volunteer in the −B and −A groups. We found that four patients (16%) showed no variation between Q1 and Q3 after three weeks. In total, 52% of the patients in the intervention group showed varying degrees of improvement.

We observed that, three weeks after the blocks, two patients in the control group (10%) were in the +A group; two (10%) in the +B group; three (15%) in the +C group, and three (15%) in the +D group; whereas three patients (15%) were in the −D group; one (5%) in the −C group; two (10%) in the −B group; and one (5%) in the −A group. We found that three patients (15%) showed no variation in their questionnaire scores. In all, 50% of the control group showed varying degrees of improvement.

**Figure 4:**
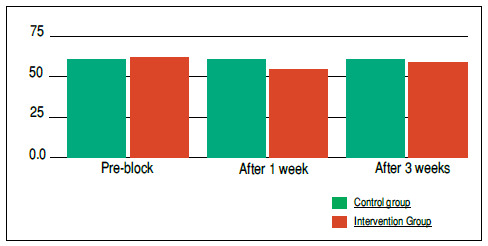
Comparison between pre-block means and those one and three weeks after the procedure for the control and intervention groups

Pre-block means according to the Oswestry index in the intervention group totaled 61.68 (± 10.4), whereas in the control group, 60.3 (± 15.3) (Figure 4).

## DISCUSSION

Lumbar disc herniation shows a greater prevalence in men, occurring mainly between the third and fifth decades of their lives.^4^ ^),(^ ^5^ ^),(^ ^7^ ^),(^ ^8^ This study found a mean age of 50.23 years and that women (53.3%) composed most participants affected by this pathology. Although our age results agree with the literature, we found a female majority, which may suggest a population profile in our sample. Souza et al.^13^ evaluated 61 patients who underwent transforaminal blocks, most of which were women (55.7%).

Cardoso et al.^14^ found that the group with the highest prevalence of herniated discs consisted of domestic service workers, as in our research. We found that the group of construction workers showed no relation with a diagnosis of disc herniation but Daltaban et al.,^15^ describe such occupation as one of the most closely related to lumbar disc herniation, agreeing with our sample. The most common location for the L5-S1 procedure (53.3%) in this study agrees with Garcia et al.,^7^ evincing the importance of the association of occupation with lumbar disc herniations and indicating that more details about them should be addressed, such as the workload.

This study shows that patients subjected to transforaminal blocks with corticosteroid and anesthetic injections experienced immediate relief but that this effect failed to continue to satisfy patients after three weeks, whether they noticeably improved or not. The control group showed a slight pain relief one week after the procedure, which failed to last after three weeks. Our comparison between group means shows no satisfactory response in any group.

Souza et al.,^13^ performed foraminal blocks in 61 patients, 32 of which with an anesthetic and corticosteroids and 29 with only a saline solution. This study observed a statistically significant improvement in the group that received medication in relation to the control group after one week according to the used pain scale and after three weeks following the Oswestry questionnaire. The control group in our study received an anesthetic with distilled water, eliciting a response that resembled that in the group that received corticosteroids, from which we can infer that improvements in the first week probably stem from the effect of the anesthetic. The improvement we observed after three weeks according to the Oswestry questionnaire indicates that we should extend our follow-up to at least three months so we can obtain more accurate and comparable results.

Manchikanti et al.’s^16^ double-blind clinical trial with 120 patients with unilateral sciatica performed transforaminal infiltrations with corticosteroids and anesthetics in one group and with anesthetics and a saline solution in the other group. Both showed significant short- and long-term improvements in relation to control. When compared to our study, this result also favors the possibility that improvements in the first weeks is related to the use of anesthetics in the solution rather than to corticosteroids.

## CONCLUSION

Results suggest the positive effect of the solution containing corticosteroids and anesthetics and that containing distilled water and anesthetics one week after we performed foraminal blocks. However, three weeks after the procedure, the corticosteroid and anesthetic solution showed no effective improvements, whereas patients blocked with distilled water and anesthetic continued to show some improvement. We should stress that the clinical picture of pain due to nerve compression has a self-limited character and may influence symptomatic variations over time.
